# Identification of Predictors of Shift Work Adaptation and Its Association With Immune, Hormonal and Metabolite Biomarkers

**DOI:** 10.1111/jpi.70017

**Published:** 2024-12-17

**Authors:** Barbara N. Harding, Ana Espinosa, Gemma Castaño‐Vinyals, Oscar J. Pozo, Debra J. Skene, Mariona Bustamante, Maria Mata, Ruth Aguilar, Carlota Dobaño, Valentin Wucher, José Maria Navarrete, Patricia Such Faro, Antonio Torrejón, Manolis Kogevinas, Kyriaki Papantoniou

**Affiliations:** ^1^ College of Population Health University of New Mexico Albuquerque New Mexico USA; ^2^ ISGlobal Barcelona Spain; ^3^ UPF Barcelona Spain; ^4^ CIBERESP Madrid Spain; ^5^ Hospital del Mar Research Institute Barcelona Spain; ^6^ Chronobiology, Faculty of Health and Medical Sciences University of Surrey Guildford UK; ^7^ CIBER de Enfermedades Infecciosas (CIBERINFEC) Barcelona Spain; ^8^ Centre for Genomic Regulation, Barcelona Institute of Science and Technology Barcelona Spain; ^9^ MeLiS, SynatAc Team, UCBL1—CNRS UMR5284—Inserm Lyon France; ^10^ French Reference Center on Paraneoplastic Neurological Syndrome, Hospices Civils de Lyon Lyon France; ^11^ University of Lyon, Université Claude Bernard Lyon 1 Lyon France; ^12^ Seat Barcelona Spain; ^13^ Department of Epidemiology, Center for Public Health Medical University of Vienna Vienna Austria

**Keywords:** adaptation, aMT6s, biomarkers, melatonin, night shift work, rotating shift work, sleep

## Abstract

We explored predictors of shift work adaptation and how it relates to disease risk biomarker levels. These analyses included 38 male, rotating shift workers, sampled twice at the end of a 3‐week night shift and a 3‐week day shift rotation. Participants collected all 24‐h urine voids, wore activity sensors, and responded to questionnaires during each shift. Using cosinor analysis, we derived the main period of urinary 6‐sulfatoxymelatonin (aMT6s) production. Adaptation was defined as the overlap between the main aMT6s production period and sleep period assessed with actigraphy. We used linear models to identify predictors of adaptation to each shift and assessed associations between adaptation profiles and hormone, cytokine, and metabolite biomarker levels. The median duration of overlap (adaptation) was 3.85 h (IQR 2.59–5.03) in the night and 2.98 (IQR 2.17–4.11) in the day shift. In the night shift, a later chronotype (coeff: −1.16, 95% CI −1.87, −0.45) and increased light at night (coeff: −0.97, 95% CI −1.76, −0.18) were associated with poorer adaptation, while longer sleep duration was associated with better adaptation (coeff: 0.46, 95% CI 0.04, 0.88). In the day shift, later sleep onset was associated with worse adaptation (coeff: −0.06, 95% CI −0.12, −0.01), while longer sleep duration was associated with better adaptation (coeff: 0.54, 0.26, 0.81). Results suggest higher androgen and inflammatory marker levels and lower levels of several metabolite markers among less adapted individuals. Chronotype, sleep, and light at night were all associated with night or day shift adaptation. Given the small sample size, results should be viewed as exploratory, but may inform interventions to optimize adaptation of rotating shift workers.

## 1 Introduction

In humans, daily physiological and behavioral rhythms are coordinated by the “master clock” located in the hypothalamic suprachiasmatic nuclei (SCN) [1] and are synchronized to the 24‐h natural light–dark cycle [2]. However, most shift workers experience misalignment between endogenous circadian rhythms and their atypical sleeping schedule [3], due to exposure to light during the biological night [2, 4], and the misaligned timing of many other integral inputs/zeitgebers (e.g., meals) [5, 6, 7], which influence entrainment of the circadian system [8, 9]. This dysregulation has been associated with a range of negative health outcomes [3].

While those who are able to adapt to their shifts may avoid some of the harmful effects of night shift work and subsequent circadian misalignment, previous research suggests that extended periods of night shift work may be required to achieve (partial) adaptation [10, 11]. In addition, rotating shift schedules may hinder adaptation compared to permanent shift work [12]. Several studies have shown that the central circadian pacemaker is not able to rapidly adjust to large phase shifts of the light–dark cycle and associated sleep‐wake cycle. Instead, experimental [13, 14] and observational [15, 16] field studies have demonstrated that endogenous rhythms driven by the SCN are slow to adapt to shift work schedules, and it is harder to phase advance than to phase delay [17]. In contrast, peripheral clocks may be quicker or slower to adapt, resulting in different degrees of internal misalignment between the SCN and peripheral clocks in many biological systems [8]. Overall, there is little existing knowledge of predictors of adaptation as well as downstream health effects (positive or negative) of shiftwork (mal)adaptation that could inform recommendations to support adaptation.

This study explored individual differences in rotating shift work adaptation using urinary 6‐sulfatoxymelatonin (aMT6s) as a central SCN circadian clock biomarker along with sleep. We also examined how adaptation to night and day shifts is related to levels of steroid hormones, immune markers and metabolite markers to provide mechanistic evidence linking shift work maladaptation to disease risk. We expected that greater levels of adaptation would lead to less perturbed biomarker levels.

## 2 Materials and Methods

### 2.1 Population

Participants were backward rotating shift workers from the automotive industry in Barcelona, Spain, who participated in the HORMONIT study [18]. The study population is described elsewhere in more detail [4]. In summary, 56 male participants were enrolled in the study, and after excluding those missing aMT6s data (*n* = 12) and those missing actigraphy data (*n* = 6), 38 participants were included in the present analyses. Workers rotated through 3 weeks of night shifts (22:00–06:00 h), 3 weeks of evening shifts (14:00–22:00 h) and 3 weeks of early morning shifts (06:00–14:00 h). Workers worked Monday to Friday with the weekend off. Participants were sampled twice; once during the 2nd or 3rd week of a night shift and again once during the 2nd or 3rd week of a day shift rotation. Participants collected all urine voids over 24‐h, wore several ambulatory sensors including (1) a HOBO monitor, HOBOware, Onset Computer Corporation, which is a small (5.8 × 3.3 × 2.3 cm), lightweight (18 g) device which measured illuminance in the range of 0.0–320 000 lux, and (2) an Actigraph 2 GT3X+, USA [19], responded to questionnaires, and donated blood at the start and end of each shift.

All participants signed an informed consent form before participating. The HORMONIT study protocol was reviewed and approved by The Parc de Salut Mar Clinical Research Ethics committee (#2015/6351).

### 2.2 Shift Work Adaptation Index

Urinary aMT6s concentrations, the major melatonin metabolite, were measured in sequential natural urine voids across a 24 h period from all participants. As explained elsewhere [4], aMT6s values were creatinine standardized (ng aMT6s/mg creatinine). We then applied cosinor analysis to plot the aMT6s rhythm across the 24‐h shift collection period and extract the peak time (acrophase) and the 24‐h average level (mesor) of production [4]. The main period of aMT6s production was defined separately for the night and day shifts as the interval at which urinary aMT6s levels rose to 75% of their peak (MEL75%up) until aMT6s levels fell to 75% of their peak (MEL75%down). This was similar to the method described by Lammers‐van der Holst et al. [20] except we used a 75%–75% range of aMT6s (instead of a 25%–25% range of melatonin from blood samples collected every 30 min), due to the wider spread of aMT6s production observed in our field study, with an average of 7 urine voids per 24 h per participant. Additionally, we collected data on sleep onset and offset times through actigraphy devices worn during the 24‐h shift collection periods. These devices measured triaxial information to assess sleep movement. The overlap (in hours) between the period of main aMT6s production and the period of main sleep episode was calculated for each participant during the night shift and separately for the day shift period. This overlap was used as a measure of adaptation, with more hours of overlap indicating better adaptation. Furthermore, we categorized our cohort into those appearing less versus more adapted during each shift period using the median hours of the overlap as the cut‐off with those with above median hours of overlap considered better adapted.

### 2.3 Predictors and Correlates of Shift Work Adaptation

We considered several variables as possible predictors of adaptation, basing this selection largely on review articles [21], or original research articles [6, 22], which examined factors related to circadian rhythm disruption. Some of these variables described more stable individual characteristics including participant age, chronotype (defined as the mid‐sleep time on a free day (MSF) after a day shift corrected for the oversleep on free days using an adapted version of the MCTQ shift questionnaire [23]), BMI (kg/m^2^), education level, smoking status (never, former, current), and duration (years) of night shift work history. In addition, we considered several variables which described habits that occurred just before or during each of the sampling periods including: shift‐specific dietary habits (breakfast/lunch/dinner time, fast length, and whether people eat during the biological night [22:00–07:00 h], with the fast length measured between the last reported meal/snack of the wakefulness period and the first meal/snack of the next wakefulness period); number of days into the 21‐day (or night) shift cycle; number of consecutive days working since the weekend; aMT6s acrophase (from cosinor analysis) from the preceding shift period (e.g., in predicting night shift adaptation, the aMT6s acrophase from the preceding day shift was incorporated); recent light exposure [from the HOBO sensor: we replaced the 0 values with 5.4 lux, which is the half of the limit of detection for the sleep periods (including main sleep and nap periods), and we set 0 values to missing for the rest of the day] including the median light levels during the early morning period (06:00–09:00 h), median light levels during 24 h, median light levels during the night (22:00–07:00 h); sleep duration (actigraphy); self‐reported sleep problems (insomnia, insufficient sleep, poor quality sleep, difficulty falling sleep, awake very late, waking up very early, feeling sleepy or tired when waking up, and whether sleep medication(s) were used) total step count during 24 h (actigraphy); sleep onset (actigraphy); smoking habits, coffee, alcohol, or medication ingestion in the 24 h before the sampling period.

In addition, we assessed how night shift work adaptation was associated with the levels of several biomarkers in our study including specific steroid hormones (11‐oxoandrosterone/11‐oxoetiocholanolone and 16‐cysteinylprogesterone), immune markers (TNFa, IL2R, IL1RA, IL4), and metabolite markers (arginine, glutamine, kynurenine, lysoPCaC18:2, lysoPCaC20:3, PCaaC34:2, PCaeC38:5), as alterations in these markers have previously been shown among night shift workers [4, 24, 25]. The analytical methods used for measuring levels of urinary hormones and blood immune and metabolite markers have been previously described elsewhere [4, 24, 25].

### 2.4 Statistical Analysis

We summarized the characteristics of the whole study population and for shift work adaptation subgroups (more vs. less adapted). Next, we graphically described individual, shift‐specific patterns in melatonin production, sleep duration and light exposure. Then, we investigated which predictors were associated with night shift adaptation (defined as the overlap between the main aMT6s production period and sleep during the night shift) and, separately, which predictors were associated with day shift adaptation (defined as the overlap between the main aMT6s production period and sleep during the day shift). To focus on the most relevant predictors to include in subsequent multiple linear regression models, we selected only those variables which were associated (*p* < 0.20) with the adaptation variables in unadjusted single regression models (results not shown). Then, we applied multiple linear regression models, adjusted for age and daylight minutes (using values corresponding to the latitude 41° 230 N and longitude 2° 100 E for Barcelona) [26], to determine which predictors were associated with differences in adaptation during the night shift period, and during the day shift period (examined in separate models). We also examined the selected predictors in single regression models adjusting for confounders (age and daylight minutes).

Finally, we assessed the mean levels of multiple steroid hormones, immune markers and metabolite markers in individuals appearing more versus less adapted to their night or day shift schedule and performed paired *t*‐tests.

We conducted a sensitivity analysis to examine the predictors of adaptation using an altered definition of the main period of aMT6s production including the time when urinary aMT6s levels rose to 50% of their peak until they fell to 50% of their peak (instead of 75%–75%). We also conducted an analysis adjusting for the week of sampling, as some participants were sampled on the 2nd and others in the 3rd week of the rotation, and this could bear an influence on the adaptation. All analyses were conducted in STATA.

## 3 Results

**Figure 1:**
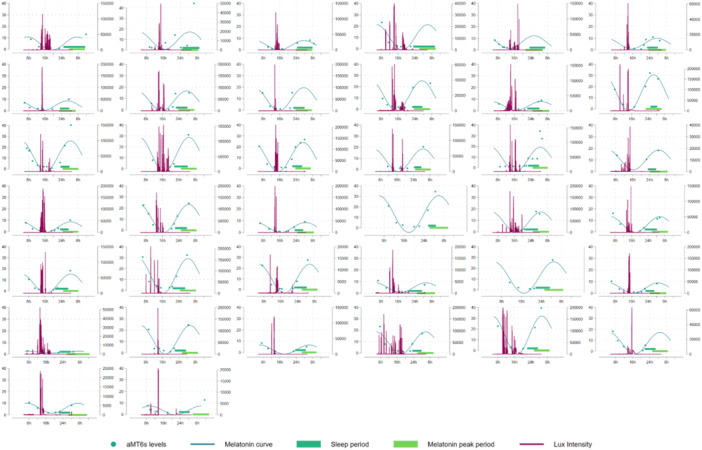
Day shift plots of aMT6s level (left side of *y*‐axis), light exposure level (lux, right side of *y*‐axis), and sleep duration (*x*‐axis) in overlapping plots for each individual. In this figure, the plots have been ordered, presenting in order those with the greatest level of adaptation first, and those with the poorest adaptation at the end. Two participants were missing data on light exposure.

**Figure 2:**
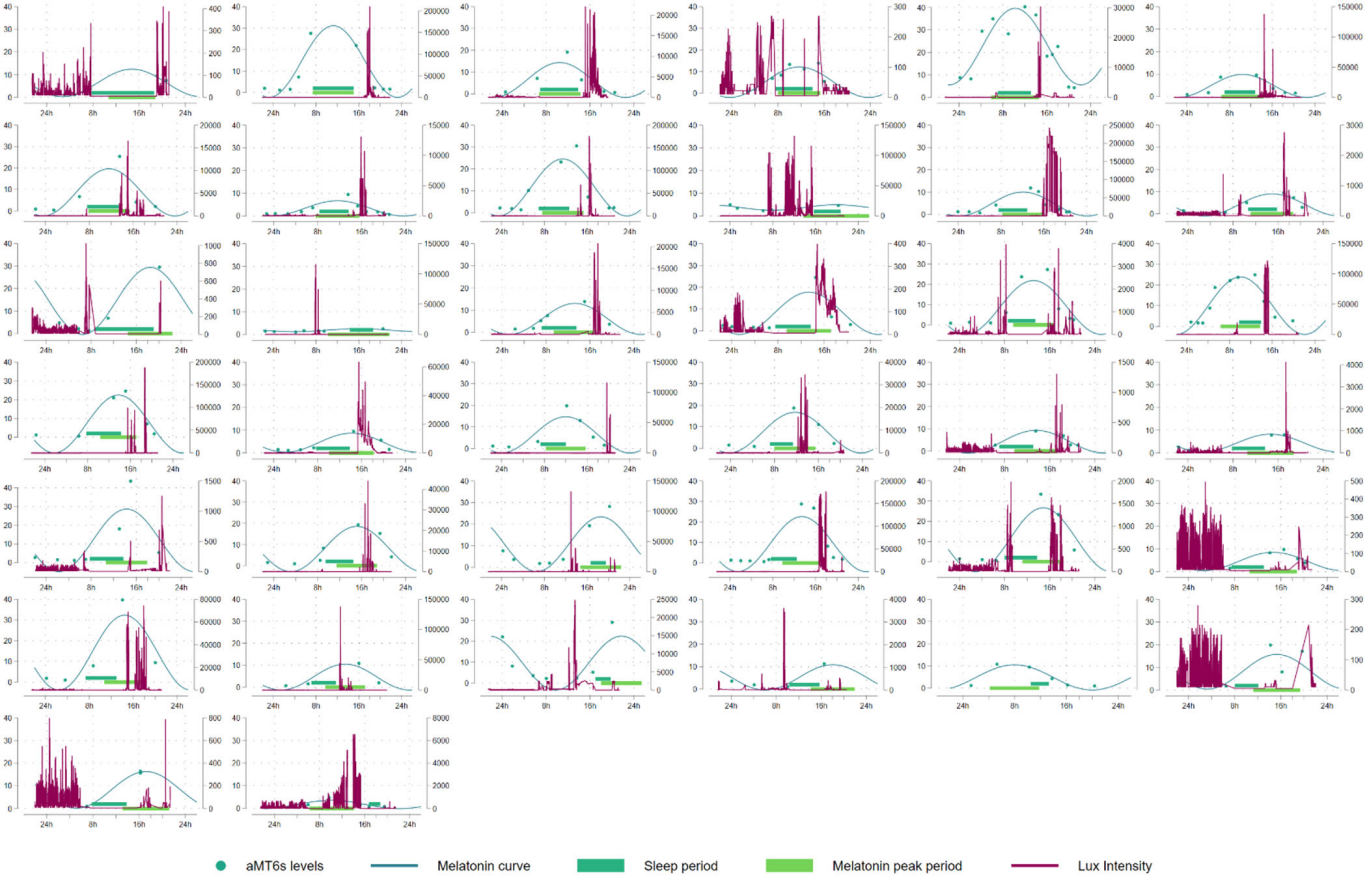
Night shift plots of aMT6s level (left side of *y*‐axis), light exposure level (lux, right side of *y*‐axis), and sleep duration (*x*‐axis) in overlapping plots for each individual so the relationship between these three variables could be examined. In this figure, the plots have been ordered, presenting in order those with the greatest level of adaptation first, and those with the poorest adaptation at the end. One participant was missing data on light exposure.

Participants were, on average, 41 years of age (IQR 34–44). The majority had a BMI of 25 kg/m^2^ or above, and 60% of the population were current or former smokers. More than half of the workers reported three or more sleep problems as well as eating during the night (22:00–07:00 h) on both their day and night shifts, and workers had been engaged in shift work for a median of 8 (IQR 5–16) years. Additional data on meal timing, sleep and light exposures are included in Table 1, and additional data on mid‐sleep times for the three chronotype groups are included in Table S1. During the night shift period, the median hours of overlap between the main aMT6s production period and the main sleep period were 3.85 h (IQR 2.59–5.03), while in the day shift period it was 2.98 (IQR 2.17–4.11). When we investigated participants who were more versus less adapted during each shift period (cut‐off of 3.85 h for night and 2.98 h for day), those who appeared more adapted during the night shift period were more likely to have never smoked, had been working shifts for fewer years, had more consecutive night shifts leading up to the sampling period, had a longer habitual night time fast when on night shifts and had less light exposure during the night compared to those who were less adapted during the night shift period (Table 1). Meanwhile, those who were more adapted to the day shift period had a higher BMI, were less likely to have a morning chronotype, had been working shifts for more years, and were more likely to fast at night (Table 1). Figures 1 and 2 show the individual variability in night and day shift work adaptation, respectively, with continuous light exposure also provided.

**Table 1:** Characteristics of study population based on night and day shift adaptation profiles.^a^

		Night shift		Day shift	
Characteristic	Full population (*n* = 38)	Less adapted (*n* = 19)	More adapted (*n* = 19)	Less adapted (*n* = 19)	More adapted (*n* = 19)
Age (years)	41 (34–44)	38 (35–46)	42 (31–44)	41 (33–46)	40 (34–44)
BMI (kg/m^2^)	26 (23–29)	26 (23–28)	26 (23–29)	24 (22–30)	26 (23–28)
MSF^b^ (HH:MM)	4:24 (3:42–4.54)	4:24 (3:42–4.54)	44:24 (3:42–5:06)	4:24 (3:42–5:12)	4:24 (3:42–4:48)
Chronotype^c^					
Morning	34%	32%	36%	42%	26%
Neither	34%	36%	32%	26%	42%
Evening	32%	32%	32%	32%	32%
Smoking					
Never	40%	37%	42%	39%	40%
Former	22%	21%	16%	28%	22%
Current	38%	42%	42%	33%	38%
Sleep complaints during work days of night shift^d^					
< 3	45%	42%	53%	37%	53%
3–5	37%	37%	37%	37%	37%
> 5	18%	21%	10%	26%	10%
Eat at night (night shift)	54%	56%	52%	47%	61%
Eat at night (day shift)	66%	63%	68%	68%	63%
Cumulative duration shift work (years)	8 (5–16)	11 (6–13)	6 (5–16)	7 (5–14)	13 (5–16)
Consecutive shifts since weekend (night shift)	2 (2–3)	2 (2–3)	3 (2–4)	2 (2–3)	3 (2–4)
Consecutive shifts since weekend (day shift)	4 (3–4)	3 (2–4)	4 (3–5)	3 (2–4)	4 (3–5)
Day of sampling night shift^e^	16 (16–17)	16 (10–17)	17 (16–18)	16 (16–17)	16 (10–18)
Day of sampling day shift^e^	17 (16–18)	17 (16–18)	18 (17–19)	17 (16–18)	18 (17–19)
Sleep duration (night shift, h)	5.8 (5.1–6.6)	5.8 (5.1–6.7)	5.8 (5.1–6.6)	5.7 (5.1–6.6)	5.9 (5.0–6.9)
Sleep duration (day shift, h)	5.8 (5.1–6.6)	5.8 (5.1–6.2)	5.8 (4.7–7.6)	5.9 (5.1–6.7)	5.7 (4.8–6.3)
Fast length (night shift, h)	12.5 (11–14.5)	11.3 (9.8–13)	13 (11.5–15)	12 (10.5–18)	12.5 (11–13.8)
Fast length (day shift, h)	8.6 (7.5–11.5)	9.0 (7.3–10.5)	8.3 (7.5–12.5)	8.5 (7.3–10.5)	9.0 (7.5–12.5)
Median light during night (night shift, lux)	43 (32–75)	65 (32–97)	43 (22–53)	48 (32–75)	43 (32–75)
Median light during night (day shift, lux)	5 (5–6)	5 (5–6)	5 (5–6)	5 (5–6)	5 (5–6)

^a^ Values are % for categorical variables and median (IQR) for continuous variables.

^b^ MSF (in HH:MM) was defined as the mid‐point of sleep on free days following a day shift corrected for oversleep on a free day.

^c^ Chronotype assessed using the MCTQ‐shift questionnaire which equates the mid‐sleep time on free days corrected for oversleep on free days following the day shift.

^d^ Sleep problems were self‐reported responses to eight domains assessing insomnia, insufficient sleep, poor quality sleep, difficulty falling sleep, awake very late, waking up very early, feeling sleepy or tired when waking up, and whether sleep medication(s) were used.

^e^ Number of days into the 21‐day (or night) shift cycle when sampling took place.

A selection of variables was ultimately included in our multiple linear regression models which examined predictors of adaptation during each shift. For the night shift, the variables included were chronotype (MSF continuous), duration of night shift work history, bedtime, fast length, median light during the night, sleep duration, and 24‐h step count. For the day shift, the variables included were bedtime, lunchtime, number of cups of coffee ingested in 24 h, sleep duration, number of days into a 21‐day shift cycle, number of consecutive days working since the weekend, and 24‐h step count. We found that during the night shift, later chronotype (coeff: −1.16, 95% CI −1.87, −0.45), and more light exposure during the night (coeff: −0.97, 95% CI −1.76, −0.18) were associated with worse adaptation, while longer sleep duration was associated with better adaptation (coeff: 0.46, 95% CI 0.04, 0.88) (Table 2). For the day shift period, a later bedtime was associated with worse adaptation (coeff: −0.06, 95% CI −0.12, −0.01), while longer sleep duration was associated with better adaptation (coeff: 0.54, 0.26, 0.81) (Table 2). For most predictors of night and day shift adaptation, results of multiple linear regression models were in line with those from adjusted single regression models in terms of direction and significance of the associations (Table S2).

**Table 2:** Predictors of night and day shift adaptation (continuous) using multiple linear regression models^a^: Coefficients and 95% CIs.

Predictors of adaptation	Coefficient^b^	95% CI lower	95% CI upper
Night shift			
Later chronotype (MSF)^c^	−1.16	−1.87	−0.45
Longer cumulative duration of shift work (years)	−0.04	−0.13	0.04
Later time of sleep onset (h)	−0.04	−0.26	0.18
Longer fast length (h)	0.12	−0.04	0.27
Greater median light during the night (lux)	−0.97	−1.76	−0.18
Longer sleep duration	0.46	0.04	0.88
Greater 24‐h step count (per 1000)	−0.04	−0.21	0.14
Day shift			
Later time of sleep onset (h)	−0.06	−0.12	−0.01
Later lunchtime	0.01	−0.24	0.27
More cups of coffee ingested in 24 h	0.15	−0.14	0.43
Longer sleep duration	0.54	0.26	0.81
More days into 3‐week rotation	0.05	−0.17	0.27
Greater number of consecutive shifts prior	0.36	−0.18	0.9
Greater 24‐h step count (per 1000)	−0.10	−0.23	0.03

^a^ Negative coefficients indicate that as the predictor increases in value, it is associated with poorer adaptation or less overlap between the main melatonin production period (75%–75%) and the sleep period.

^b^ Models include all potential predictors and additionally adjust for age and daylight minutes.

^c^ MSF was defined as the mid‐point of sleep on free days following a day shift corrected for oversleep on free days.

When assessing the levels of other biomarkers during each work shift in relation to the adaptation in the respective work shift, only the level of kynurenine was statistically significantly higher during the night shift period for those with better adaptation profile (3.15 vs. 2.54 µM, *p* = 0.03) (Table 3). However, we observed suggestive patterns of higher levels of inflammatory markers (IL2R and IL1‐RA) in worse adaptation to night shift (Table 3), higher levels of hormones (11‐oxoandrosterone/11‐oxoetiocholanolone and 16‐cysteinylprogesterone) (Table 4) and lower levels of several metabolite markers (Arginine, Glutamine, Kynurenine, LysoPCaC18:2, and PCaeC38:5) during both shifts among those with worse adaptation (Table 3).

**Table 3:** Mean levels of cytokines and metabolite markers based on night and day shift adaptation profiles.^a^

Analyte	Night shift (mean (SD))			Day shift (mean (SD))		
	Less adapted	More adapted	*p* value^b^	Less adapted	More adapted	*p* value^b^
Cytokines and chemokines^c^						
TNFa	4.5 (5.0)	4.6 (5.4)	0.95	6.0 (5.3)	4.6 (5.5)	0.44
IL2R	143 (78)	142 (118)	0.98	161 (98)	151 (103)	0.76
IL1RA	608 (305)	533 (503)	0.58	884 (544)	792 (434)	0.58
IL4	7.5 (8.6)	16.2 (27.1)	0.19	20 (32.2)	8.3 (5.9)	0.12
Metabolite markers^d^						
Arginine	103 (31)	111 (38)	0.52	92 (21)	103 (17)	0.10
Glutamine	39 (18)	46 (31)	0.41	39 (19)	40 (16)	0.70
Kynurenine	2.5 (0.7)	3.2 (0.9)	0.03	2.6 (0.8)	2.9 (0.7)	0.23
LysoPCaC18:2	83 (25)	82 (30)	0.87	62 (17)	68 (32)	0.46
LysoPCaC20:3	4.8 (1.5)	4.5 (1.8)	0.54	3.9 (1.2)	4.1 (1.3)	0.64
PCaaC34:2	158 (20)	158 (23)	0.98	146 (17)	147 (29)	0.91
PCaeC38:5	14 (3)	15 (4)	0.40	14 (3)	15 (3)	0.46

Abbreviations: IL1RA, interleukin‐1 receptor agonist; IL2R, interleukin‐2 receptor; IL4, interleukin 4; SD, standard deviation; TNFa, tumor necrosis factor alpha.

^a^ Using data from blood samples collected at 6:00 h from the day shift (start of shift) and at 6:00 h from the night shift (end of shift).

^b^ *p* values were calculated from two‐sample *t*‐tests.

^c^ Units pg/mL.

^d^ Units μM/mL.

**Table 4:** Total production, acrophase, and mesor of hormones based on night and day shift adaptation profiles.^a^

Hormone production characteristics	Night shift (mean (SD))			Day shift (mean (SD))		
	Less adapted	More adapted	*p* value^b^	less adapted	more adapted	*p* value^b^
Total production^c^						
11‐oxoandrosterone/11‐oxoetiocholanolone^d^	12 764 (11758)	8234 (10647)	0.22	7108 (6758)	5141 (2403)	0.24
16‐cysteinylprogesterone^e^	426 (1156)	36 (30)	0.15	223 (655)	519 (2092)	0.56
Acrophase^f^						
11‐oxoandrosterone/11‐oxoetiocholanolone^d^	14:56 (5 h 31 min)	14:28 (7 h 5 min)	0.49	14:23 (7 h 1 min)	11:47 (5 h 55 min)	0.22
16‐cysteinylprogesterone^e^	17:24 (4 h 37 min)	14:11 (1 h 15 min)	0.04	8:05 (3 h 17 min)	10:05 (5 h 37 min)	0.19
Mesor^g^						
11‐oxoandrosterone/11‐oxoetiocholanolone^d^	531.9 (489.9)	343.1 (433.6)	0.22	296.2 (281.6)	214.2 (100.2)	0.24
16‐cysteinylprogesterone^e^	17.8 (48.2)	1.5 (1.3)	0.15	9.3 (27.3)	21.6 (87.2)	0.56

^a^ Using data from natural urine voids over 24‐h collection periods.

^b^ *p* values were calculated from two‐sample *t*‐tests.

^c^ The total production indicates 24 h levels (ng metabolite/mg creatinine).

^d^ 11‐oxoandrosterone/11‐oxoetiocholanolone is an androgen.

^e^ 16‐cysteinylprogesterone is a progestogen.

^f^ The acrophase indicates the time of peak hormone production expressed in clock time.

^g^ The mesor indicates the mean level of 24 h production of the hormone.

Results from the sensitivity analysis, which examined an alternative adaptation definition that included a wider interval of main aMT6s production (50%–50% of aMT6s peak production instead of 75%–75%), remained largely similar (Table S3). The same possible predictors plus a few additional ones (BMI, breakfast time, lunch time, number of caffeinated sodas ingested in 24 h, and average light during early morning 06:00–09:00 h) reached statistical significance *p* < 0.20 with the altered definition of the adaptation variable during the night shift in single linear regression models and were included in the night shift multiple linear regression predictor model, however, none were statistically significantly associated with night shift adaptation. Similar predictors were also included in the day shift adaptation model, and a later bedtime and shorter sleep duration remained associated with less adaptation, as shown in the primary models (Table S3). Finally, we present the results from day and night shift adaptation models, which were adjusted for the week of sampling (Table S4) and were similar to the main model results.

## 4 Discussion

The ability of an individual to adapt to rotating shift work and align their biological rhythms to the constantly changing working schedule is important for worker health and safety. In a real‐world study among rotating night shift workers in a slow (3‐week) backward rotation system, we found variability in the adaptability to the different shifts and identified some predictors of this adaptability. Specifically, we found that later chronotype, more light exposure during the night, and shorter sleep duration were associated with worse adaptation in the night shift period, while later sleep onset and shorter sleep duration were associated with worse adaptation in the day shift period. Biomarker levels such as sex hormones, immune markers, and metabolites appeared at different levels among shift workers who were better adapted to their shifts.

We defined adaptation considering the hours of overlap between the main period of aMT6s production and the main sleep episode to reflect the extent of alignment of the central SCN clock with the sleep/wake cycle. This novel approach has been used in one other study [20]. Several other studies have focused solely on a marker of endogenous circadian rhythms [27, 28, 29] or solely on sleep characteristics [30] to determine adaptation. Furthermore, the majority of research to date on adaptation has been conducted in tightly controlled laboratory‐based simulation studies which did not incorporate real‐world shift working populations as we have done here.

In our examination of predictors of adaptation, we examined predictors of both night and day shift adaptation separately. This was done because the day shift values are not really a reference, given that our population of rotating shift workers is changing their working hours every 3 weeks. In fact, in our study, the mean period of overlap between the main aMT6s production period and main sleep episode was longer in the night shift period (3.85 h) than in the day shift period (2.98 h), indicating poor adaptation to early morning shifts following night shifts in a slow backward rotation system (every 3 weeks).

We found that sleep timing, sleep duration, light at night, and chronotype predicted shift work adaptation in our population. Sleep habits, age, chronotype, and genetic susceptibility may influence the ability to adapt to night shift work. Prior research has established a link between sleep duration and quality and circadian adaptation to night shift work [31]. Although higher shift work adaptation was associated with better sleep quality and fewer sleep problems, shift workers in our population were sleep deprived in both shifts, achieving on average less than the recommended 7–9 h of sleep for adults, and the majority had three or more sleep problems. These results suggest that more focus be placed on addressing sleep issues among shift workers, with an emphasis on providing the most scientific evidence to support good sleep hygiene and to encourage adequate sleep duration. The association between later sleep onset and worse adaptation during the night shift may be explained by later sleep onset leading to shorter sleep (and perhaps also poorer sleep) as well as poorer adaptation, or later sleep onset may result in more morning light exposure or more artificial light exposure at night and a resulting suppression or delay of the aMT6s rhythm and as a consequence a reduced overlap of melatonin production and sleep episode [32]. In fact, our results also showed that higher night‐time light exposure was associated with poorer adaptation, but in our study, workers were exposed to relatively low light levels during the night shift (< 100 lux). Higher light exposure during the night shift has been associated with suppression of nocturnal melatonin production, aMT6s acrophase delay and circadian disruption related to the process of re‐entrainment of the aMT6s rhythm [2]. Light may have important implications in studies of circadian adaptation and shift work tolerance, and administrating (or preventing) light at given times assists in improving nighttime alertness and reducing the sleepiness of shift workers [33]. However, in a review of six studies among permanent night shift workers, only a small minority underwent a complete phase adjustment of their endogenous melatonin, and light intensity did not influence shift work adaptation [10]. For chronotype, we found that later chronotype assessed using an adapted version of the MCTQ‐shift questionnaire (information was available for night shifts, day shifts, and respective days off but not evening shifts or days off following evening shifts) was associated with worse adaptation during the night shift, which was at odds with our expectations. And, this finding was not statistically significant in the single regression models. Most studies found that morning chronotypes have more difficulties adapting to night shift work than evening types as measured by problems with sleep [12]. However, these findings were not adjusted for other predictors of adaptation, were in some cases based on different instruments for chronotype assessment, did not use measures of circadian phase to define adaptation, or did not study night work adaptation in conjunction with adaptation to the day shift in a rotating shift schedule [21]. It is possible that given the specific rotating schedule of our participants, including very early morning shifts and a slow backward rotation, later chronotypes may not fare as well.

Our findings show that those who were less adapted during both shift rotations appeared to generally have higher androgen levels (and for the night shift also higher progestogen levels), higher levels of multiple inflammatory markers (IL2R and IL1‐RA), and lower levels of several metabolite markers (Arginine, Glutamine, Kynurenine, and PCaeC38:5). While the exact mechanisms underlying these findings are not known, the observation that some steroid hormones were elevated among individuals with a poorer adaptation profile may be relevant in cancer development or progression, as adrenal‐derived androgens have been identified as key components in the development and progression of castration‐resistant prostate cancer [34, 35]. In addition, progesterone receptors have been confirmed in several cancers, including prostate cancer [36]. Furthermore, multiple immune markers were elevated among participants with a poorer adaptation during the day shift. These inflammatory markers have a role in the pathogenesis of chronic diseases, for example, cardiovascular disease [37] and cancer [38]. Additionally, we found that several metabolite markers were lower among individuals with worse adaptation. The perturbation of metabolic pathways is related to a variety of health outcomes [8], although the pathways involved are diverse and numerous and should be further explored.

A crucial question is whether it is beneficial for rotating night shift workers to adapt their biological rhythms to align with their work and sleep schedules and to what extent, given they will have to rotate back to a different schedule [27]. The answer surely depends in part on the speed and direction of the rotation, individual characteristics that determine the ability to phase shift sleep and internal rhythms but also on whether adaptation is related to improved health and sleep outcomes. The present study found evidence to suggest that analyte levels driven by peripheral clocks were less perturbed among those who were well‐adapted. This, however, should be examined further in additional studies, which can also adequately address the connection between biological adaptation and shift work tolerance [21, 39].

This study had several strengths, including the access to biomarker data on sequential urinary aMT6s collected across 24‐h, as well as detailed data on sleep from actigraphy. An additional strength is the inclusion of data on a selection of hormones, immune, and metabolite biomarkers. Furthermore, we had repeated measures for all participants and were able to examine daily rhythms and adaptation during both the night and day shift periods. This study is the first of its kind to address the predictors of adaptability on a night shift and back to a day shift among rotating night shift workers. It is also the first to our knowledge to examine the role of shift work adaptability on such a wide range of biomarkers. There are, however, some limitations of the present study, including the small sample size, and the focus only on male subjects, which limits the generalizability of the findings. In addition, some of our participants were sampled in the 2nd week while others were sampled in the 3rd week, which could, on its own, explain some of the variability in adaptation. Also, we were unable to examine how quickly our participants shifted their rhythms, given that we only sampled them once during each shift rather than several days in a row during each of the shift periods. An additional limitation is that urinary aMT6s acrophase approximates the circadian phase and is likely masked by light exposure and physical movement. Dim light melatonin onset (DLMO) is a more sensitive marker of the endogenous circadian phase that could be used in future studies to define adaptation or circadian entrainment, however its application in field studies of shift workers is challenging. Although we did not have DLMO data, MCTQ‐based chronotype assessment has been validated against behavioral and physiologic measures of the circadian phase in populations of non‐shift workers [40, 41]. And some of these studies have shown that chronotype from the MCTQ‐based chronotype assessment predicts DLMO accurately, even when sleep schedules are irregular [23, 42]. An additional concern is that the day shift of our population starts very early (06:00 h), which may be challenging for many of our participants to adapt to and may be accentuated by cultural norms in Spain surrounding rather late meal timings, sleep timings, and social activities. Finally, this study did not examine psychosocial factors or family responsibilities as predictors for adaptation. We did not collect data on factors such as time commitments, family arrangements, stress, and others, which would be interesting to examine in a future study.

In conclusion, within our population of slow, backward‐rotating shift workers, we observed that a later chronotype, more light at night, and shorter sleep duration were associated with poorer adaptation during the night shift rotation, while later sleep onset and shorter sleep duration were associated with poorer adaptation during the day shift rotation. We found evidence that biological adaptation defined using a central circadian clock biomarker approach was associated with changes in biomarkers of disease risk.

## Author Contributions

Barbara N. Harding, Ana Espinosa, and Kyriaki Papantoniou contributed to the study design. Gemma Castaño‐Vinyals, Manolis Kogevinas, José Maria Navarrete, Patricia Such Faro, and Antonio Torrejón contributed to data acquisition. Barbara N. Harding, Ana Espinosa, Oscar J. Pozo, Debra J. Skene, Ruth Aguilar, Carlota Dobano and Valentin Wucher contributed to data analysis. Barbara N. Harding, Ana Espinosa, Gemma Castaño‐Vinyals, Mariona Bustamante, Manolis Kogevinas, and Kyriaki Papantoniou contributed to the interpretation of data. Barbara N. Harding and Kyriaki Papantoniou contributed to the writing of the paper. All authors provided a critical review of the manuscript.

## Conflicts of Interest

José Maria Navarrete, Patricia Such Faro, and Antonio Torrejón work at the Occupational Health service of the car factory, which was the setting of the present study. Within the HORMONIT study working group, they express their own views and do not represent the company. The other authors declare no conflicts of interest.

## Supporting information

Supporting information.

## Data Availability

The data that support the findings of this study are available on reasonable request from the corresponding author. The data are not publicly available due to privacy and ethics restrictions.
