# Supplementary material for: Identification of Predictors of Shift Work Adaptation and Its Association With Immune, Hormonal and Metabolite Biomarkers
Source: J Pineal Res. 2024 Dec 17;76(8):e70017. doi: 10.1111/jpi.70017 (PMC11664228; doi:10.1111/jpi.70017)
Supplement: Supplementary file 1 — Supporting information. [file JPI-76-e70017-s001.docx]

**Supplemental Material.**

Supplemental Table 1: Description of mid-sleep time on free days, in the three chronotype groups (morning, neither, evening) determined with the Munich Chronotype Questionnaire for shift workers (MCTQ-shift).

|  | Mid-sleep time on free days (MSF)^a^ | | | |
| --- | --- | --- | --- | --- |
| Chronotype  groups | Mean | Min | Max | Variance (minutes) |
| Morning | 3:29 | 2:44 | 3:49 | 5 |
| Neither | 4:25 | 4:03 | 4:47 | 2 |
| Evening | 5:37 | 4:49 | 8:08 | 55 |

^a^MSF was defined as the mid-point of sleep on free days following a day shift corrected for oversleep on free days, expressed in clock time

Supplemental Table 2. Predictors of night and day shift adaptation (continuous) using simple linear regression models: coefficients and 95% CIs

| Predictors of adaptation^a^ | Coefficient^b^ | 95% CI lower | 95%CI upper |
| --- | --- | --- | --- |
| NIGHT SHIFT |  |  |  |
| Later chronotype (MSF)^c^ | -0.50 | -1.13 | 0.13 |
| Longer cumulative duration of shift work (years) | -0.03 | -0.12 | 0.06 |
| Later time of sleep onset (h) | -0.19 | -0.40 | 0.01 |
| Longer fast length (h) | 0.09 | -0.10 | 0.28 |
| Greater median light during the night (lux) | -1.10 | -1.97 | -0.24 |
| Longer sleep duration | 0.52 | 0.16 | 0.88 |
| Greater 24-hour step count (per 1000) | -0.14 | -0.33 | 0.05 |
| DAY SHIFT |  |  |  |
| Later time of sleep onset (h) | -0.12 | -0.19 | -0.05 |
| Later lunchtime | 0.25 | -0.06 | 0.57 |
| More cups of coffee ingested in 24 hours | 0.29 | -0.07 | 0.64 |
| Longer sleep duration | 0.75 | 0.46 | 1.03 |
| More days into 3-week rotation | 0.29 | 0.05 | 0.52 |
| Greater number of consecutive shifts | 0.86 | 0.37 | 1.36 |
| Greater 24-hour step count (per 1000) | -0.17 | -0.35 | 0.01 |

^a^Negative coefficients indicate that as the predictor increases in value, it is associated with poorer adaptation or less overlap between the main melatonin production period (75%-75%) and the sleep period.

^b^Models are adjusted for age and daylight minutes

^c^MSF was defined as the mid-point of sleep on free days following a day shift corrected for oversleep on free day

Supplemental Table 3. Predictors of night and day shift adaptation, using a modified definition of adaptation^a^: coefficients and 95% CIs

|  | Coefficient^b^ | 95% CI lower | 95%CI upper |
| --- | --- | --- | --- |
| **NIGHT SHIFT** |  |  |  |
| Later chronotype (MSF)^c^ | -0.61 | -1.89 | 0.68 |
| Higher Body Mass Index (BMI) | 0.14 | -0.14 | 0.42 |
| Longer cumulative shift work duration (years) | -0.02 | -0.17 | 0.13 |
| Later time of sleep onset (h) | -0.24 | -0.72 | 0.25 |
| Later breakfast time | 0.04 | -0.42 | 0.49 |
| Later lunch time | -0.04 | -0.89 | 0.82 |
| Higher number of caffeinated sodas ingested in 24 hours | 0.39 | -0.82 | 1.60 |
| Greater median light in early morning (6-9 h) | 0.03 | -0.03 | 0.09 |
| Greater median light at night (22-7 h) | -1.29 | -2.79 | 0.21 |
| sleep duration | 0.32 | -0.75 | 1.40 |
| Greater 24-hour step count (per 1000) | -0.17 | -0.56 | 0.23 |
| **DAY SHIFT** |  |  |  |
| Later time of sleep onset (h) | -0.08 | -0.13 | -0.02 |
| Later breakfast time | -0.01 | -0.20 | 0.19 |
| Later lunch time | -0.02 | -0.27 | 0.23 |
| More days into 3-week rotation | 0.08 | -0.15 | 0.31 |
| Higher number of consecutive shifts | 0.39 | -0.14 | 0.92 |
| Longer sleep duration | 0.57 | 0.30 | 0.85 |

^a^Negative coefficients indicate poorer adaptation or less overlap between the main melatonin production period (50%-50%) and the sleep period.

^b^Models include all potential predictors and additionally adjust for age and daylight minutes

^c^MSF was defined as the mid-point of sleep on free days following a day shift corrected for oversleep on free days

Supplemental Table 4: Predictors of night and day shift adaptation (continuous) using multiple linear regression models, additionally adjusted for week of sampling^a^: coefficients and 95% CIs

|  | Original results | | | Results adjusting for week of sampling | | |
| --- | --- | --- | --- | --- | --- | --- |
| Predictors of adaptation | Coefficient^b^ | 95% CI lower | 95%CI upper | Coefficient^b^ | 95% CI lower | 95%CI upper |
| NIGHT SHIFT |  |  |  |  |  |  |
| Later chronotype (MSF)^c^ | -1.16 | -1.87 | -0.45 | -1.08 | -1.82 | -0.34 |
| Longer cumulative duration of shift work (years) | -0.04 | -0.13 | 0.04 | -0.05 | -0.13 | 0.04 |
| Later time of sleep onset (h) | -0.04 | -0.26 | 0.18 | -0.04 | -0.26 | 0.18 |
| Longer fast length (h) | 0.12 | -0.04 | 0.27 | 0.13 | -0.03 | 0.29 |
| Greater median light during the night (lux) | -0.97 | -1.76 | -0.18 | -1.00 | -1.80 | -0.20 |
| Longer sleep duration | 0.46 | 0.04 | 0.88 | 0.49 | 0.06 | 0.92 |
| Greater 24-hour step count (per 1000) | -0.04 | -0.21 | 0.14 | -0.03 | -0.21 | 0.15 |
| DAY SHIFT |  |  |  |  |  |  |
| Later time of sleep onset (h) | -0.06 | -0.12 | -0.01 | -0.06 | -0.11 | 0.00 |
| Later lunchtime | 0.01 | -0.24 | 0.27 | -0.02 | -0.26 | 0.23 |
| More cups of coffee ingested in 24 hours | 0.15 | -0.14 | 0.43 | 0.14 | -0.13 | 0.40 |
| Longer sleep duration | 0.54 | 0.26 | 0.81 | 0.59 | 0.32 | 0.86 |
| More days into 3-week rotation | 0.05 | -0.17 | 0.27 | -0.97 | -2.35 | 0.41 |
| Greater number of consecutive shifts prior | 0.36 | -0.18 | 0.9 | 1.30 | -0.04 | 2.64 |
| Greater 24-hour step count (per 1000) | -0.10 | -0.23 | 0.03 | -0.11 | -0.24 | 0.01 |

^a^Negative coefficients indicate that as the predictor increases in value, it is associated with poorer adaptation or less overlap between the main melatonin production period (75%-75%) and the sleep period.

^b^Models include all potential predictors and additionally adjust for age, daylight minutes, and week of sampling (whether collected during week 2 or during week 3 of the rotation).

^c^MSF was defined as the mid-point of sleep on free days following a day shift corrected for oversleep on free day.
